# Amplicon_sorter: A tool for reference‐free amplicon sorting based on sequence similarity and for building consensus sequences

**DOI:** 10.1002/ece3.8603

**Published:** 2022-03-01

**Authors:** Andy R. Vierstraete, Bart P. Braeckman

**Affiliations:** ^1^ Laboratory of aging physiology and Molecular Evolution University of Gent Gent Belgium

**Keywords:** amplicon sequencing, biodiversity, consensus, DNA barcoding, metabarcoding, metagenetics, Oxford Nanopore Technologies, replacing Sanger

## Abstract

Oxford Nanopore Technologies (ONT) is a third‐generation sequencing technology that is gaining popularity in ecological research for its portable and low‐cost sequencing possibilities. Although the technology excels at long‐read sequencing, it can also be applied to sequence amplicons. The downside of ONT is the low quality of the raw reads. Hence, generating a high‐quality consensus sequence is still a challenge. We present Amplicon_sorter, a tool for reference‐free sorting of ONT sequenced amplicons based on their similarity in sequence and length and for building solid consensus sequences.

## Graphical abstract

Oxford Nanopore sequencing is low‐cost sequencing technology capable of sequencing amplicons. Hence, generating a high‐quality consensus sequence is still a challenge. Amplicon_sorter is a tool for reference‐free sorting of ONT sequenced amplicons based on their similarity in sequence and length and for building solid consensus sequences.

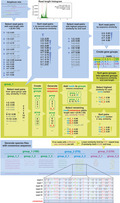

## 1 INTRODUCTION

Long‐read sequencing methods from Oxford Nanopore Technologies (ONT) (Eisenstein, 2012) can also be used to mass sequence amplicons. In comparison with short‐read sequencers such as Illumina (2 × 300 bp) and IonTorrent (600 bp) (Slatko et al., 2018), there is virtually no limit to the amplicon length for ONT. However, to this date, the main disadvantage of ONT is the relatively low read quality, which most recently reached a modal of 99.3% with the new Q20+ technology and an R10.4 flow cell (https://nanoporetech.com/accuracy).

Many ONT applications and tools exist (Wang et al., 2021), but specific tools for processing and consensus calling of amplicon sequences are limited. Several programs and pipelines are available to create a consensus sequence based on existing reference sequences (Krehenwinkel et al., 2019; Maloney et al., 2020; Moore et al., 2020; Sikolenko & Valentovich, 2021; Strassert et al., 2021). Reads of mixed samples (soil, water, food, feces…) containing sequences of species not yet included in databases can be difficult to be assigned to a species or genus (Wei et al., 2020) with standard Operational Taxonomic Unit (OTU) clustering programs (Bolyen et al., 2019; Rognes et al., 2016a; Schloss et al., 2009). Unknown species may be assigned to incorrect genera because of the high error rate in the reads and low similarity with available sequences. This may result in the generation of a consensus sequence based on a mixture of the sequences of two or more species. To analyze amplicons and come to a consensus without the availability of reference sequences, several steps have to be performed. Reference‐free consensus sequences have been made before to identify bacteria (Calus et al., 2018; Davidov et al., 2020; Karst et al., 2021; Rodríguez‐Pérez et al., 2021), viruses (Chan et al., 2020), fungi (Morrison et al., 2020; Simmons et al., 2020), invertebrates (Chang et al., 2020; Knot et al., 2020), and vertebrates (Pomerantz et al., 2018; Seah et al., 2020) or to replace Sanger sequencing by ONT consensus methods (Simmons et al., 2020). Most of these analyses perform the four following steps: 1. Barcoded reads are demultiplexed while basecalling in Guppy or afterward with the guppy_barcoder in the Guppy suite (https://community.nanoporetech.com), Porechop (https://github.com/rrwick/Porechop), Minibar (Krehenwinkel, Pomerantz, Henderson, et al., 2019), qcat (https://github.com/nanoporetech/qcat), or by using UMIs (Karst et al., 2021). 2. A quality filtering step based on quality scores and length can be added by using NanoFilt (de Coster et al., 2018), seqtk (https://github.com/lh3/seqtk), PRINSEQ (https://github.com/uwb‐linux/prinseq), or fastp (Chen et al., 2018). 3. The reads are clustered and a consensus is made with Canu (Koren et al., 2017), MAFFT (Katoh & Standley, 2013), vsearch (Rognes et al., 2016b), IsONclust (Sahlin & Medvedev, 2020), or Consension (https://microbiology.se/software/consension). 4. In most cases, a last consensus polishing step is performed with Medaka (https://github.com/nanoporetech/medaka), Racon (Vaser et al., 2017), Nanopolish (Loman et al., 2015), or a reading frame correction for coding genes (Menegon et al., 2017; Srivathsan et al., 2018, 2021a, 2021b).

Most current pipelines (Maestri et al., 2019; Menegon et al., 2017; Srivathsan et al., 2018) need these consecutive programs to demultiplex, sort amplicons based on length/species identity with references to finally create a consensus sequence. IsoCon and ToFu are reference‐free long‐read consensus algorithms for transcriptome data that have been described but aim for a different application (Gordon et al., 2015; Sahlin et al., 2018). The recent programs ONTrack (Maestri et al., 2019), NGSpeciesID (Sahlin et al., 2021), and ONTbarcoder (Srivathsan et al., 2021a) perform reference‐free clustering of amplicons and create a high‐quality consensus sequence and are designed for specific amplicon sequencing applications. ONTrack needs demultiplexed files, processes only the reads in the most abundant cluster, and needs the large fast5 files to polish the consensus sequence. NGSpeciesID processes demultiplexed files with one or a few divergent amplicons. It only needs a fastq file as input and clusters the sequences based on similarity. A preferred amplicon length and deviation thereof can be entered in the script. ONTbarcoder is specifically made to process COI amplicons that are uniquely tagged with a barcode. It needs a demultiplexing file which contains the unique barcode‐primer sequences, a fastq file with the sequences, and the expected fragment length. Although it expects one amplicon per unique barcode, it can find divergent amplicons (even other genes) with the same length if more are present. Here we present Amplicon_sorter which is developed to sort sequences based on similarity and length, and to build a robust consensus sequence for each group of sequences in one simple run. Amplicon_sorter can process all sorts of amplicons, with or without barcode unlike ONTbarcoder that processes coding genes and needs a barcode for each sample. Amplicon_sorter and NGSpeciesID can process a range of amplicon lengths in one go unlike ONTbarcoder that need one expected fragment length. Amplicon_sorter does not limit the search to the most abundant clusters like ONTrack and ONTbarcoder but searches for everything. Unlike ONTrack and NGSpeciesID which are pipelines that are dependent on other programs to do the job, Amplicon_sorter is a python script that only needs python3 and a few python plugins. Amplicon_sorter might perform even better in some cases in conjunction with Medaka, but this is in most cases not needed. It has been written for metagenetics samples that contain amplicons of several genes with the same or different lengths from all the species in the samples. Nevertheless, it can also be used for demultiplexed samples that only contain one amplicon.

## 2 SOFTWARE DESCRIPTION

### 2.1 Installation and dependencies

Amplicon_sorter is available at https://github.com/avierstr/amplicon_sorter. The script is written in Python 3 and depends on a few third‐party Python modules: c‐implementation of Levenshtein (https://pypi.org/project/python‐Levenshtein), super‐fast library for sequence alignment edlib (Šošić & Šikić, 2017), Biopython (Cock et al., 2009), and Matplotlib (Hunter, 2007). It runs on Linux/Unix/MacOSx platforms and uses multiprocessing. One GB ram per used core is sufficient for data analyses.

### 2.2 Workflow

#### 2.2.1 Gene group creation

**FIGURE 1:**
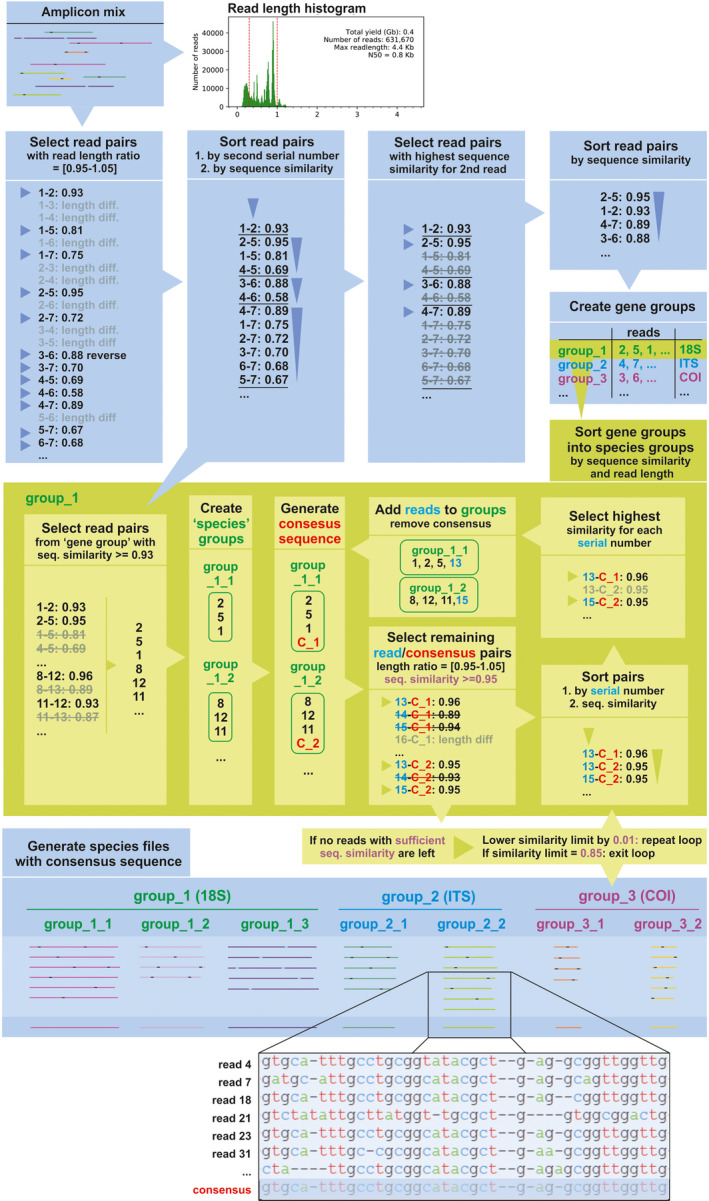
A step‐wise schematic diagram of the workflow of Amplicon_sorter

The Amplicon_sorter script reads the input file in fasta or fastq format (Figure 1). Prior to analysis, minimum and maximum read lengths can be delimited and the maximum number of sampled reads for analysis can be set. In absence of a user limit, Amplicon_sorter will analyze 10,000 reads by default. If the number of reads in the input file is lower than 1000, all reads are used. All reads get a unique serial number. An option (‐a ‐‐all) is available to compare all reads with each other, but this is discouraged for sequence sets of over 100,000 reads because it is computation intensive. For example: on a 3.8 GHz system with 16 cores, comparing 100,000 reads with the ‐‐all option takes 116 h user time (8 h 35 min real time), 8× random sampling the total number of reads without the ‐‐all option takes only 18 h user time (2 h 20 min real time).

Without this option, the script subsamples the selected number of reads in batches of 1,000 in the same order as the reads in the inputfile (an option (‐ra ‐‐random) is available to randomly sample from the inputfile) and compares the reads pairwise within each batch for read length differences smaller than 5%. If the similarity is lower than 50%, the reverse complement of one of the sequences is also compared. If the similarity is greater than or equal to 80%, the serial numbers of the two compared sequences and their similarity is added to a list. This list is saved to disk for later use (step 2.2.2). Next, for each read, only the read to which it has the highest similarity is kept resulting in a high‐similarity pair. Gene groups are created by merging high‐similarity pairs with overlapping reads. It may occur that, eventually, several gene groups remain that actually represent the same gene. To combine those, Amplicon_sorter samples 50 random reads from each group, creates a consensus, and compares the consensus of each group with each other. A length difference of 8% is allowed, and if the similarity is greater than or equal to 60%, the gene groups are merged. The script saves the result in gene group files that contain reads of the same gene based on length and similarity (e.g., group_1 contains 18S reads, group_2 contains COI reads, etc.).

#### 2.2.2 Gene‐to‐species sorting

Unlike Tofu and IsoCon which use a nearest neighbor graph method to cluster the reads, Amplicon_sorter uses a more straightforward approach. For each read, only the read to which it has the highest similarity is retained. Gene‐to‐species sorting is an iterative process within each gene group, starting with grouping reads with high sequence similarity (greater than or equal to 93%) into species groups. Species groups that contain common sequences are merged. A consensus sequence for each species group is built to which all remaining sequences in the gene group are compared with a maximum length difference of 5%. A sequence is added to the species group to which it has the highest similarity (of at least 95%). A new consensus is built after each iteration and therefore becomes increasingly more accurate. When no more reads can be added (or a limit of 3 cycles for the same similarity), the similarity threshold is dropped by 1% and a new cycle starts. Every other cycle, the consensuses from all species groups that have a maximum length difference of 8% are compared. If the similarity between two consensuses is greater than or equal to 96%, the two species groups are merged. When the similarity threshold dropped to 85%, the loop ends and the sequences from each species group are saved in a file. This iterative process converges to a stable point in each iteration but is limited to 3 cycles because adding more cycles increases the processing time and is only marginally improving the consensus in that cycle. As a result, each output file contains all sequences with high similarity and similar length as well as a final consensus sequence based on 200 random reads (e.g., file_1_1.fasta is 18S from species1, file1_2.fasta is 18S from species2, file_2_1 is COI from species 3 …). Amplicon_sorter generates extra files containing all consensus sequences per species group and a list of all consensus sequences in the project. Reads that could not be grouped are saved in “unique sequences” files. The script allows for parallel processing to speed up the analysis. Output files can be saved in fasta and fastq format. Amplicon_sorter writes and reads temporary files to keep the RAM memory consumption low.

## 3 EXAMPLES AND COMPARISON WITH SIMILAR TOOLS

### 3.1 Parameter optimization

**FIGURE A1:**
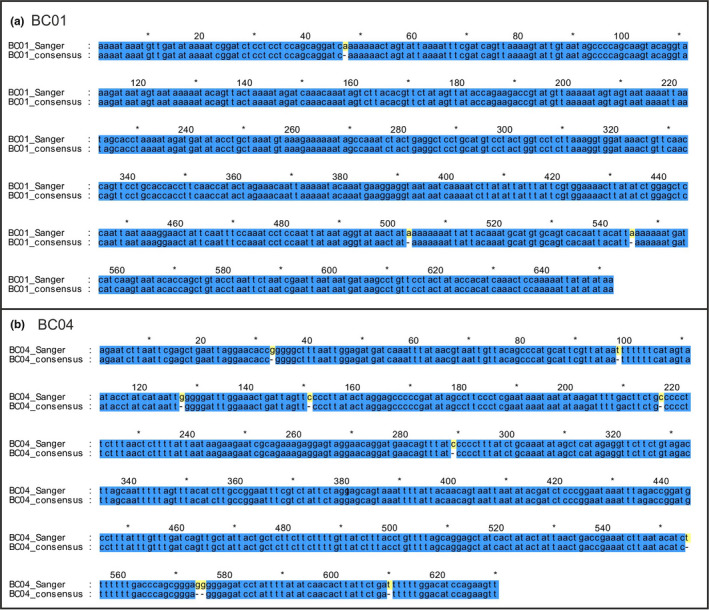
Comparison between the Sanger sequence and the consensus produced by Amplicon_sorter. (a) BC01 with 99.54% similarity. (b) BC04 with 98.57% similarity. Errors are consistent underestimations of homopolymer length by ONT basecalling

**FIGURE A2:**
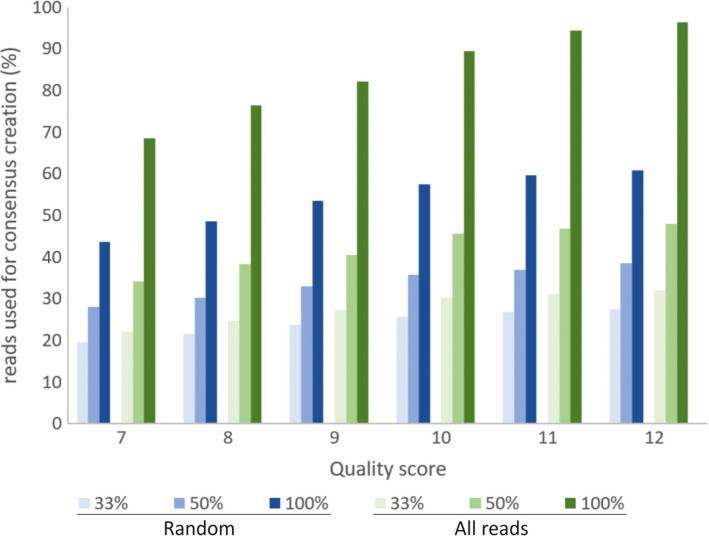
The percentage of reads used to create a consensus plotted against the read quality score. Blue: randomly sampled reads with the default settings of Amplicon_sorter. Green: comparing all reads with each other (requires more processing time)

An online available amplicon dataset sequenced on an R9.4 MinION flow cell (Maestri et al., 2019) was used to optimize the parameters of Amplicon_sorter. The dataset contains barcoded amplicons from two snails and five beetles with similarities ranging from 69% to 89% (Table A1). To test the maximal consensus accuracy of Amplicon_sorter in a single species, the script was run on the separate barcode files. Consensus accuracies were reached ranging from 98.41% to 99.54% and all errors were homopolymer underestimations (Figure A1). After pooling all seven barcode samples and creating input files with quality score between Q7 and Q12 using NanoFilt, we were able to retrieve all original barcodes from all input files (Table 1). The low abundant barcodes (BC04: 7.6%, BC07: 3.2%) were detected in the Q7 input file and even the lowest abundance of 1.5% from BC07 was detected in the Q12 input file. If very low abundance barcodes should be found, we recommend using the ‐‐all option (to compare all reads with each other) at the cost of processing time. An alternative option to find very low abundance barcodes is to use the random function ‐‐random and increase the number of comparisons ‐‐maxreads to a number higher than the available reads. This way the program samples reads randomly several times to increase the chances to find its best match (example command for Q12 33% reads: python3 amplicon_sorter.py ‐i poolded_q12.fastq ‐o q12_30 ‐min 600 ‐max 800 ‐np 10 ‐maxr 13062) (‐i = input file, ‐o = output folder, ‐min = minimum read length, ‐max = maximum read length, ‐np = number of cores, ‐maxr = maximum number of reads to use). Lower quality reads are less likely to be assigned to a group (Table A2, Figure A2). The percentage of all reads within a barcode that were used to create the consensus is shown. For this concatenated dataset of 7 barcodes, the “random” setting of the program was used. Because by default Amplicon_sorter samples several times 1000 reads from the input file, some reads are selected multiple times from the pool while others are never selected. This results in an average of 60% of reads that are used for consensus creation per barcode when sampling 100% of the number of reads from the high‐quality pool. When sampling the low‐quality pool, only 43% of the reads are recovered. When choosing the “compare all” option, there are no duplicate reads in the comparison. This results in 68% on average for the low‐quality dataset to 96% for the high‐quality reads. We can conclude that Amplicon_sorter has a high sorting and recovery capability for the reads in the sample.

**TABLE 1:** Amplicon_sorter analyses on the pooled dataset of Maestri et al. (2019). For the quality scores Q7, Q9, and Q12, the percentage of reads in the pool is shown. For each barcode (BC), the consensus accuracy is shown. Several rounds were performed with random sampling of 33%, 50%, and 100% of the reads

	BC01	BC02	BC03	BC04	BC05	BC06	BC07
Q7							
% reads in pool	9.4	24.7	7.7	7.6	19.9	27.5	3.2
33% reads sampled	99.39	99.43	99.10	98.41	99.14	99.00	99.44
50% reads sampled	99.23	99.15	99.26	98.57	99.28	99.00	99.25
100% reads sampled	99.54	99.29	99.26	98.25	99.14	99.00	99.25
Q8							
33% reads sampled	99.23	99.86	98.95	98.25	99.28	99.00	99.25
50% reads sampled	99.54	99.29	98.95	98.57	99.28	99.00	99.25
100% reads sampled	99.39	99.29	98.95	98.57	99.28	99.00	99.25
Q9							
% reads in pool	10.2	25.5	8.3	6.2	20.6	26.6	2.6
33% reads sampled	99.39	99.15	99.11	98.57	99.28	99.00	99.44
50% reads sampled	99.39	99.29	99.11	98.41	99.28	99.00	99.44
100% reads sampled	99.54	99.15	99.26	98.41	99.14	99.00	99.25
Q10							
33% reads sampled	99.54	99.29	99.41	98.57	99.28	99.00	99.25
50% reads sampled	99.54	99.29	99.11	98.57	99.28	99.00	99.44
100% reads sampled	99.54	99.29	99.26	98.76	99.42	99.00	99.44
Q11							
33% reads sampled	99.54	99.57	98.81	98.57	99.28	99.00	99.44
50% reads sampled	99.23	99.43	99.11	98.57	99.28	99.00	99.25
100% reads sampled	99.54	99.43	99.11	98.57	99.14	99.00	99.44
Q12							
% reads in pool	17.4	30.0	9.6	4.4	19.7	17.4	1.5
33% reads sampled	99.54	99.43	99.26	98.57	99.28	99.00	99.25
50% reads sampled	99.54	99.43	99.40	98.73	99.28	99.00	99.44
100% reads sampled	99.54	99.57	99.41	98.73	99.28	99.00	99.44

### 3.2 Separation limits

To further test the potential and boundaries of Amplicon_sorter, we generated a new ONT sequence data set using a specific set of amplicons and species that allowed us to cover several questions. The first goal was to combine amplicons of up to three genes per barcode to test if Amplicon_sorter could distinguish them and how accurate the resulting consensus would be compared to the Sanger reference sequence. The second goal was to detect the separation limit of Amplicon_sorter for a given gene of closely related species. In our third goal, we wanted to test whether long amplicons can be sequenced with only a part of that amplicon being available as reference to check the consensus accuracy. Our ONT sequence data set was comprised of several barcoded amplicons (spacer and COI) from two mollusks and several insect species with similarities ranging from 85 to 100% (Tables A3, A4 and A3, A4). The Sanger sequence was available for the spacer and COI amplicons, while for the tandem repeat (last 700 bp of 18S ‐ spacer region ‐ first 1300 bp of 28S) only the spacer region was available as a reference. The amplicon test samples were sequenced with the ligation kit (SQK‐LSK109, Oxford Nanopore Technologies, UK) on a 9.4.1 MinION flowcell. Basecalling was done with Guppy v4.2.2 with the HAC (High Accuracy) option as well as with the SupHAC (Super Accuracy) option in Guppy v5.0.7.

#### 3.2.1 Amplicon_sorter, ONTBarcoder, and NGSpeciesID output for separate barcodes

In a first approach, we tested the separation limit of Amplicon_sorter, ONTbarcoder, and NGSpeciesID using our demultiplexed ONT sequence data set. Reads were selected with NanoFilt for a quality score of minimum 12 and demultiplexing was done with Minibar. Each barcode sample contained up to three genes (COI 700 bp, spacer 750 bp, and some a tandem repeat part of 2800 bp). Amplicon_sorter was able to sort the reads and build the consensus for each gene of which we had the complete Sanger sequence with an accuracy between 98.2% and 100% (Table 2). We also polished the Amplicon_sorter results with Medaka 1.4.3 for accuracy improvement. However, only 2 out of 10 consensus sequences, which had no perfect match with the Sanger reference, were improved by Medaka polishment. Despite the availability of only a short Sanger reference for the long tandem repeat, Amplicon_sorter was able to build an accurate consensus. ONTbarcoder and NGSpeciesID produced similar consensus sequences, although ONTbarcoder could not produce a consensus for BC111 and BC115 because of the low number of reads that passed the selection criteria of the program (Table 2) and the spacer sequences were cataloged as “remaining” sequences because there is no translation table approval for these noncoding amplicons in the program. ONTbarcoder had to be run several times with different expected fragment lengths to find the different genes. NGSpeciesID uses an extra polishing step with Medaka.

**TABLE 2:** Percent similarity of the Sanger reference sequence with consensus sequences generated by Amplicon_sorter, Amplicon_sorter polished with Medaka, ONTbarcoder, and NGSpeciesID

Barcode	Species and gene	Amplicon_sorter	Amplicon_sorter + Medaka	ONTbarcoder	NGSpeciesID
BC101	*On*. *boudoti* Spacer	99.8	99.8	99.8	99.7
BC102	*On*. *forcipatus* Spacer	100.0	100.0	100.0	99.8
BC103	*O*. *cecilia* Spacer	99.8	99.8	99.8	99.8
BC104	*C*. *mzymtae* Spacer	99.3	99.3	99.3	99.2
BC105	*O*. *reductus* Spacer	98.6	98.6	98.6	98.6
BC107	*C*. *buchholzi* COI	98.2	98.2	98.2	98.2
	*C*. *buchholzi* Spacer	100.0	100.0	100.0	99.8
BC108	*C*. *insignis* COI	99.8	99.8	99.8	99.8
	*C*. *insignis* Spacer	98.8	98.8	97.2	98.4
BC109	*C*. *bidentata* COI	100.0	100.0	100.0	100.0
BC110	*C*. *amasina* COI	100.0	100.0	100.0	100.0
BC111	*G*. *schneiderii* COI	99.8	100.0	95.2*	99.8
BC112	*G*. *vulgatissimus* COI	99.7	99.8	99.8	99.7
BC114	*G*. *kinzelbachi* COI	99.8	99.8	99.8	99.8
BC115	*G*. *pulchellus* COI	100.0	100.0	83.5*	96.8

Note

Maximal similarities between consensus and Sanger reference per barcode are shaded (BC = barcode, On. = *Onychogomphus*, O. = *Ophiogomphus*, C. = *Cordulegaster*, G. = *Gomphus*, * = did not pass criteria in ONTbarcoder).

#### 3.2.2 Running Amplicon_sorter and NGSpeciesID without demultiplexing the reads

A second analysis was performed without demultiplexing the reads to test the separating power of Amplicon_sorter and NGSpeciesID for closely related species. Such test was impossible for ONTbarcoder as the program requires a file with barcode and primer sequences to run. Reads were selected with NanoFilt for a minimum quality score of 12. Adapters and barcodes were removed with Porechop. In our dataset, there is a gap in similarity between 94% and 98%. Data from Srivathsan et al. (2021b) was used to fill this gap: five Diptera species were selected (Table A5) for which the Sanger reference sequences had a similarity around 95% and 96%. The raw MinION reads from dataset A (Flongle, mixed Diptera) and C (R10.3, mixed Diptera, 1 million reads) were Blasted against the reference sequences and reads from the selected species were saved in one file for each dataset. Using the default settings, Amplicon_sorter was able to distinguish species with up to 95% similarity for the High Accuracy basecalled reads from a 9.4.1 flow cell and between 95 and 96% similarity for reads from an R10.3 flow cell (Table 3). For the Super Accuracy basecalled reads and the R10.3 High Accuracy reads, the results could be improved by changing the default settings for ‐‐similar_species_groups from 93% to 94% and for ‐‐similar_consensus from 96% to 98% because of the higher accuracy of the basecaller (SupHAC) or the more accurate flow cell (R10.3). With the default settings, several species were merged. *Ophiogomphus cecilia* and *O*. *reductus* have a similarity of 98%, which does not allow species separation by the script. Therefore, it averages the consensus sequences of these and other highly similar species. NGSpeciesID was not able to distinguish species with a similarity above 90% and merged one or more species in one consensus. Changing the ‐‐rc_identity_treshold values in the range of 0.91 to 0.97 did not improve the results. Several runs were performed by changing the expected amplicon length from 600 up to 1000 bp with 50 bp increases per step. For the R10.0 data, NGSpeciesID failed to perform the polishing step with Medaka so a three times polishing with Racon was performed.

**TABLE 3:** Species separated by Amplicon_sorter and NGSspeciesID from a pooled dataset

	Amplicon_sorter		NGSpeciesID	
	HAC	SupHAC*	HAC	SupHAC
***Cordulegaster***				
*C*. *buchholzi* COI	98.2 (1099)	98.2 (1074)	–	–
*C*. *insignis* COI	99.7 (2103)	99.8 (1879)	96.3 (1424)	–
*C*. *bidentata* COI	100 (1843)	99.8 (2323)	97.9 (2066)	–
*C*. *amasina* COI	99.8 (1129)	100 (127)	–	–
***Gomphus***				
*G*. *schneiderii* COI	100 (2718)	100 (1905)	–	–
*G*. *vulgatissimus* COI	99.8 (2440)	99.8 (1916)	97.8 (1469)	97.4 (5365)
*G*. *lucasii* COI	98.6 (2566)	99.3 (1294)	97.25 (1183)	–
*G*. *kinzelbachi* COI	99.8 (1261)	99.8 (1270)	–	–
*G*. *pulchellus* COI	99.8 (1061)	100 (775)	–	–
***Onychogomphus/Ophiogomphus***				
*On*. *boudoti* Spacer	99.8 (3371)	99.8 (4626)	–	95.5 (4767)
*On*. *forcipatus* Spacer	99.7 (1809)	100 (646)	–	–
*O*. *cecilia* Spacer	99.8 (7943)	99.8 (8107)	98.5 (5743)	99.4 (5113)
*O*. *reductus* Spacer	–	–	–	–
***Cordulegaster***				
*C*. *mzymtae* Spacer	–	99.4 (201)	–	–
*C*. *buchholzi* Spacer	–	–	–	–
*C*. *insignis* Spacer	99.1 (3637)	99.3 (3765)	99.2 (4767)	99.2 (6473)
**Data Srivathsan et al.**	Flongle HAC	R10.3 HAC*	Flongle HAC	R10.3 HAC
O89399_MOD01	99.7 (153)	100 (905)	99.8 (635)	99.8 (3346)#
O89401_MOD01	–	99.7 (376)	–	–
O89376_MOD01	99.8 (92)	99.8 (118)	–	–
O89825_MOD06	99.8 (639)	100 (634)	100 (1836)	99.7 (53)#
O89486_MOD02	–	100 (183)	–	–

Note

Similarity of the consensus sequence with the Sanger reference, listed for reads basecalled with the High Accuracy and Super Accuracy basecaller. Number of reads is added between brackets. Yellow highlights indicate decreased similarity because closely related species were grouped. Maximal similarities between consensus and Sanger reference per species are shaded. (HAC: High Accuracy, SupHAC: Super Accuracy, ‐: not found, *: default settings for ‐‐similar_consensus changed from 96 to 98 and ‐‐similar_species_groups from 93 to 94, #: 3 times polished with Racon, Flongle HAC: sequenced on a flongle flowcell and basecalled with High Accuracy, R10.3 HAC: sequenced on an R10.3 flow cell and basecalled with High Accuracy).

**FIGURE A3:**
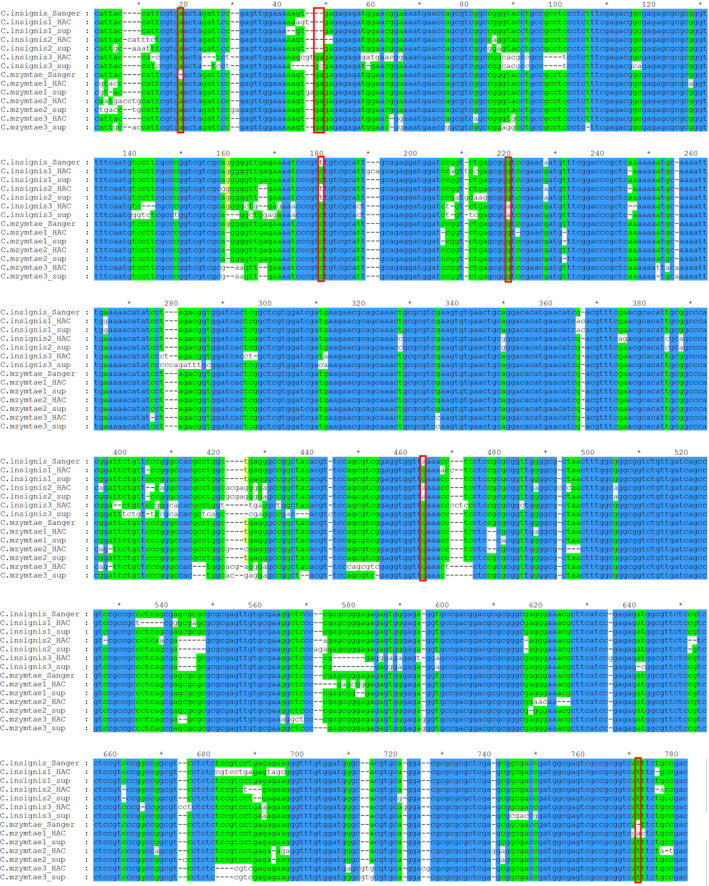
Comparison of a few raw ONT spacer reads from *Cordulegaster insignis* and *C*. *mzymtae* with the Sanger references. There are no consistent differences between *insignis* and *mzymtae* reads because of the high error rate (red rectangles in the alignment indicate the differences in Sanger sequence between both species). This is likely the reason why the script cannot separate these reads into specific groups

When considering raw reads in a group, the similarity to the Sanger reference varies between 86% and 98% (Table A6, Figure A3), which may explain the species separation limit of around 95%–96% similarity. When species with over 95% similarity occur in the same pool, the consensus sequence will have a lower similarity to the Sanger reference (if available) because of the averaging effects. If the accuracy of the basecaller will further improve, especially for homopolymer calling, this limit will likely increase.

**FIGURE A4:**
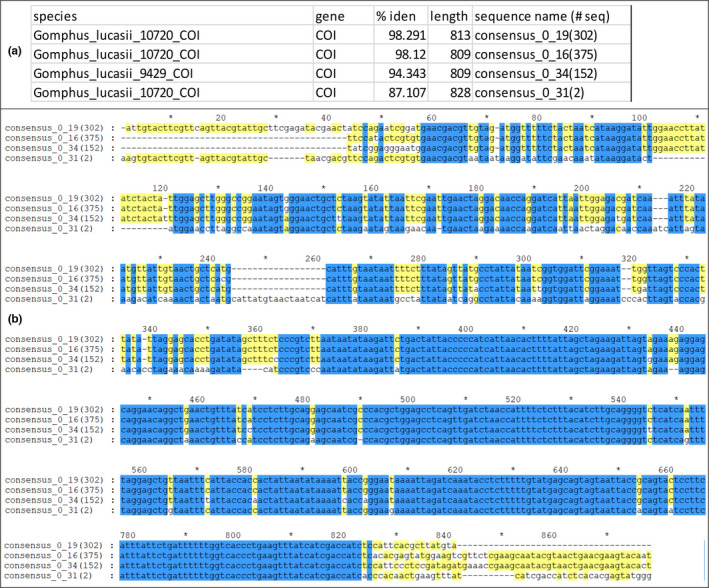
Example of false/redundant consensus sequences produced by Amplicon_sorter. (a) four consensus sequences of the same species. The first two with similar identity to the Sanger reference and more than 300 reads. The third and fourth have a much lower similarity. (b) alignment of those reads. The first consensus has 40 extra bases at the 5’ end, the second read has 40 extra bases more at the 3’ end. The middle parts of both reads are almost identical. The third sequence differs in many positions and is built from 152 reads with similar errors. The last sequence differs even more and is a consensus built from only two reads

While the other tools only search for the most abundant cluster(s), Amplicon_sorter searches for everything. As a result, for amplicons from low‐quality PCR, it may produce more/false/redundant consensus sequences than the other tools, sometimes even multiple consensus sequences for the same species. If the initial PCR is of high quality, the result should be one consensus per species. If the PCR is less successful (smear, multiple bands), Amplicon_sorter produces multiple consensus sequences (Figure A4). These can have the same similarity with the Sanger reference, but still contain an adapter or primer at one side that was missed by trimming. In case the PCR produced incomplete amplicons, also shorter consensus sequences from the same species are generated. ONT sequences are characterized by random errors, but if Amplicon_sorter finds a few reads with the same error, it will make a new consensus of these reads. These false/redundant consensus sequences are usually built from a low number of reads, contrary to the correct consensuses. The number of reads that produce a consensus is indicated between brackets in the output consensussequences.fasta file.

## 4 DISCUSSION

Amplicon_sorter creates high‐quality consensus sequences for barcoded or non‐barcoded amplicons sequenced with ONT. When compared to programs with similar purpose, the consensus sequences have a similar or higher quality which is mostly between 99% and 100%. It is remarkable that Amplicon_sorter by default is producing similar or better high‐quality consensus sequences than NGSpeciesID which uses an extra polishing step with Medaka, and ONTbarcoder which uses a genetic code translation table to correct the consensus. Polishing consensus sequences from Amplicon_sorter with Medaka barely improves their quality. The reverse side of the coin is that Amplicons_sorter is 7 times slower than NGSpeciesID and ONTbarcoder in processing the samples. A dataset with one amplicon took NGSpeciesID 2 min 30 s and Amplicon_sorter 16 min 30 s to process. Another dataset with amplicons between 600 and 950 bp took NGSpeciesID 75 min while Amplicon_sorter used 550 min user time.

**FIGURE A5:**
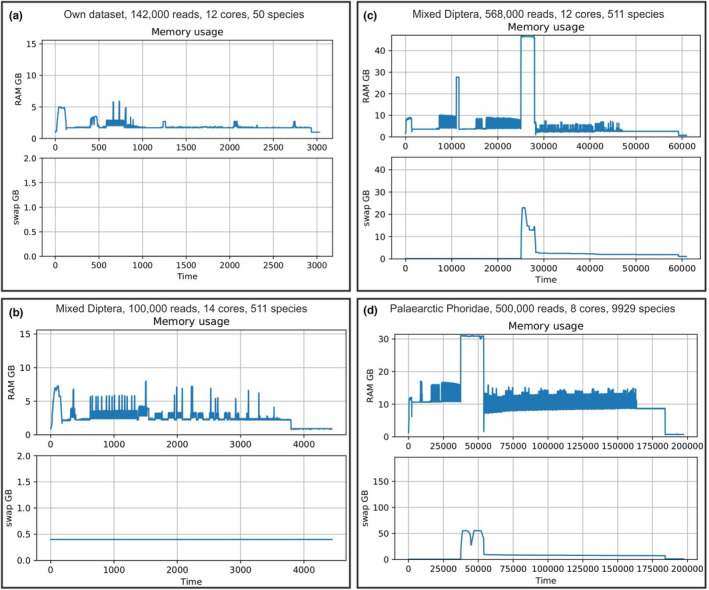
Memory utilization during a run of Amplicon_sorter when analyzing datasets with different number of species and number of reads sampled. (a) 50 species with multiple genes and 142,000 reads sampled. The memory consumption did not exceed 7 GB. (b) 511 species with one gene, 100,000 reads used. Memory consumption had a peak around 8 GB. (c) 511 species with one gene, 568,000 reads used. A peak of 70 GB when sorting species. (d) 9929 species, one gene and 500,000 reads used. Around 85 GB of memory was used to sort the species

Amplicon_sorter has been tested on two datasets (Srivathsan et al., 2021b) containing 511 and 9929 species with numbers of reads used ranging from 100,000 to 568,000 (Figure A5, Table A7). The memory usage peaked to 80 GB when creating the species groups using the highest number of reads. Using a higher number of reads is necessary to have sufficient read coverage for each species. Analyzing datasets with a large number of species is limited by the amount of available memory on the computer.

In mixed samples, Amplicon_sorter can find low abundance samples (1.5%) with the default settings, and if the option to compare all sequences with each other (‐all) is used, even lower abundance species can be recovered. This option is computation intensive and is discouraged for samples with more than 100,000 reads. By default, Amplicon_sorter compares the reads in batches of 1000 sequences with each other to speed up the process.

Amplicon_sorter outperforms NGSpeciesID and ONTbarcoder when processing metagenetic samples which contain several amplicons of the same or different length from distant or closely related species. The separating limit is around 95 or 96% depending on the type of flow cell and basecaller version used. There is no need to specifically indicate an expected amplicon length, instead a range with minimum and maximum length can be entered to search for all possible amplicons within that range.

## 5 CONCLUSION

Amplicon_sorter is an easy‐to‐use tool to group sequences to species or genus level without the need for reference sequences. It automatically creates a consensus sequence for each group of reads. It can be used for samples where only one species is present or samples with several species and genes with different lengths. The limit for separating closely related species within a sample is currently around 95%–96%.

## CONFLICT OF INTEREST

The authors declare no conflicts of interest.

## AUTHOR CONTRIBUTIONS

**Andy R. Vierstraete:** Conceptualization (lead); Data curation (lead); Formal analysis (lead); Methodology (lead); Software (lead); Validation (lead); Writing – original draft (lead); Writing – review & editing (lead). **Bart P. Braeckman:** Supervision (supporting); Writing – review & editing (supporting).

## AVAILABILITY AND IMPLEMENTATION

Amplicon_sorter is written in Python3 and released under the GNU GPL 3.0 License. The source code and documentation are available at https://github.com/avierstr/amplicon_sorter. The script is written for Linux/Unix/MacOSx and is a command line tool.

## Data Availability

The sequencing data generated for this study is available at Dryad (https://doi.org/10.5061/dryad.zgmsbccd0).

## APPENDIX 1

**TABLE A1:** Similarity between Sanger reference sequences of the amplicons from the Maestri et al. (2019) data set: pairwise similarity is shown between barcodes (BC)

	BC1	BC2	BC3	BC4	BC5	BC6	BC7
**BC1**	100%						
**BC2**	73%	100%					
**BC3**	72%	73%	100%				
**BC4**	72%	73%	84%	100%			
**BC5**	69%	70%	79%	89%	100%		
**BC6**	71%	70%	82%	81%	79%	100%	
**BC7**	72%	73%	82%	81%	81%	79%	100%

**TABLE A2:** The percentage of all reads within a barcode that are used to create the Amplicon_sorter consensus sequence depends on quality score, the fraction of total reads sampled, and sampling strategy (random sampling (R) vs all reads (A)). For quality scores Q7, Q9, and Q12, the abundance (%) of reads in each barcode pool is added. The average for all barcodes is shown in the last two columns

	BC01		BC02		BC03		BC04		BC05		BC06		BC07		Average	
	R	A	R	A	R	A	R	A	R	A	R	A	R	A	R	A
**Q7**																
% reads in pool	9.4		24.7		7.7		7.6		19.9		27.5		3.2		100.0	
33% sampled	22.2	26.8	21.1	24.0	21.8	25.0	12.1	14.9	21.3	23.9	18.8	21.7	9.3	10.5	19.6	22.2
50% sampled	31.7	38.0	30.2	37.3	31.5	37.0	17.5	21.5	30.4	36.5	26.6	33.7	13.3	15.6	28.0	34.3
100% sampled	50.4	78.6	47.4	72.9	48.8	76.5	28.8	43.7	46.5	71.3	41.4	67.6	21.1	32.9	43.7	68.5
**Q8**																
33% sampled	24.4	27.3	22.6	26.4	22.8	26.4	15.7	18.7	22.3	26.1	20.8	24.6	12.6	14.0	21.5	24.7
50% sampled	32.7	42.2	32.0	39.7	32.2	40.9	22.2	28.2	31.4	40.8	39.4	37.5	16.5	20.8	30.2	38.4
100% sampled	53.3	83.9	51.7	80.4	51.3	81.8	36.9	55.3	51.8	78.4	46.5	75.6	26.6	42.8	48.7	76.4
**Q9**																
% reads in pool	10.2		25.5		8.3		6.2		20.6		26.6		2.6		100.0	
33% sampled	25.8	30.0	24.7	28.6	24.9	28.1	19.3	22.4	23.9	27.9	23.5	26.5	14.5	16.0	23.7	27.3
50% sampled	35.7	44.6	35.0	42.0	33.6	42.6	25.8	32.1	33.8	41.7	32.8	39.2	18.7	24.6	33.1	40.5
100% sampled	57.6	88.9	56.1	85.4	56.4	84.3	42.6	67.0	55.7	83.0	51.7	81.5	32.1	48.0	53.6	82.1
**Q10**																
33% sampled	26.8	31.8	27.1	31.5	25.3	30.7	21.8	24.3	27.1	30.9	25.1	29.9	15.9	20.2	25.8	30.2
50% sampled	37.5	48.3	38.0	47.1	36.1	47.9	30.5	39.0	36.7	47.8	34.1	43.5	22.1	29.5	35.7	45.6
100% sampled	60.1	93.9	60.5	92.5	60.0	93.0	48.4	75.8	58.5	90.3	55.8	88.9	36.4	54.8	57.5	89.4
**Q11**																
33% sampled	27.7	31.9	28.1	32.5	27.5	32.8	23.5	27.5	27.9	31.8	25.7	30.3	18.9	20.4	27.0	31.3
50% sampled	38.5	47.9	37.3	48.1	38.4	46.6	32.7	40.5	38.3	49.3	36.0	45.3	22.7	31.5	36.9	46.8
100% sampled	61.8	97.2	61.6	97.3	60.4	97.5	51.4	80.0	62.7	97.6	57.3	91.7	40.0	62.6	59.7	94.4
**Q12**																
% reads in pool	17.4		30.0		9.6		4.4		19.7		17.4		1.5		100.0	
33% sampled	28.4	32.8	28.3	32.9	28.2	33.7	25.3	29.8	26.3	30.2	27.2	31.9	25.3	30.8	27.5	32.1
50% sampled	35.5	48.9	40.4	48.5	38.8	48.6	34.2	41.6	38.6	49.5	36.7	46.5	34.3	49.9	38.6	48.1
100% sampled	62.4	98.8	62.4	98.6	60.5	97.1	56.3	86.9	62.0	97.0	57.7	91.0	54.9	94.8	60.9	96.3

**TABLE A3:** Similarity of Sanger sequences between species for the spacer region (ITS1, 5.8S, ITS2): Pairwise similarity is shown between closely related species

*Onychogomphus/Ophiogomphus*	*On. boudoti*	*On. forcipatus*	*O. cecilia*	*O. reductus*
***On. boudoti***	100%			
***On. forcipatus***	92%	100%		
***O. cecilia***	89%	87%	100%	
***O. reductus***	89%	87%	98%	100%

*Cordulegaster*	*C. mzymtae*	*C. insignis*	*C. buchholzi*
***C. mzymtae***	100%		
***C. insignis***	99%	100%	
***C. buchholzi***	99%	100%	100%

**TABLE A4:** Similarity of Sanger sequences between species for COI: Pairwise similarity is shown between closely related species

*Gomphus*	*G. schneiderii*	*G. vulgatissiumus*	*G. lucasi*	*G. kinzelbachi*	*G. pulchellus*
***G. schneiderii***	100%				
***G. vulgatissiumus***	93%	100%			
***G. lucasii***	90%	89%	100%		
***G. kinzelbachi***	85%	85%	85%	100%	
***G. pulchellus***	86%	86%	86%	88%	100%

*Cordulegaster*	*C. buchholzi*	*C. insignis*	*C. amasina*	*C. bidentata*
***C. buchholzi***	100%			
***C. insignis***	94%	100%		
***C. amasina***	93%	94%	100%	
***C. bidentata***	93%	93%	93%	100%

**TABLE A5:** Similarity of Sanger sequences between species for COI (data from Srivathsan et al., 2021a): Pairwise similarity is shown between closely related species

Data from Srivathsan et al. (2021a)	O89399_MOD01	O89401_MOD01	O89376_MOD01	O89825_MOD06	O89486_MOD02
**O89399_MOD01**	100%				
**O89401_MOD01**	99%	100%			
**O89376_MOD01**	96%	96%	100%		
**O89825_MOD06**	88%	88%	88%	100%	
**O89486_MOD02**	86%	86%	86%	95%	100%

**TABLE A6:** Similarities among three random ONT reads of the Spacer gene of *Cordulegaster insignis* and *Cordulegaster mzymtae*

*Cordulegaster* HAC basecalling	*C*. *insignis* Sanger	*C*. *insignis* 1	*C*. *insigni*s 2	*C*. *insignis* 3	*C*. *mzymtae* 1	*C*. *mzymtae* 2	*C*. *mzymtae* 3	*C*. *mzymtae* Sanger
***C*** **. *insignis* Sanger**	100%							
***C*** **. *insignis* 1**	94%	100%						
***C*** **. *insignis* 2**	92%	88%	100%					
***C*** **. *insignis* 3**	87%	84%	83%	100%				
***C*** **. *mzymtae* 1**	96%	92%	90%	86%	100%			
***C*** **. *mzymtae* 2**	95%	90%	89%	84%	93%	100%		
***C*** **. *mzymtae* 3**	91%	88%	88%	83%	89%	89%	100%	
***C*** **. *mzymtae* Sanger**	99%	94%	92%	88%	97%	95%	92%	100%

*Cordulegaster* SupHAC basecalling	*C*. *insignis* Sanger	*C*. *insignis* 1	*C*. *insigni*s 2	*C*. *insignis* 3	*C*. *mzymtae* 1	*C*. *mzymtae* 2	*C*. *mzymtae* 3	*C*. *mzymtae* Sanger
***C*** **. *insignis* Sanger**	100%							
***C*** **. *insignis* 1**	98%	100%						
***C*** **. *insignis* 2**	94%	93%	100%					
***C*** **. *insignis* 3**	86%	86%	82%	100%				
***C*** **. *mzymtae* 1**	97%	97%	92%	85%	100%			
***C*** **. *mzymtae* 2**	97%	97%	92%	85%	97%	100%		
***C*** **. *mzymtae* 3**	92%	92%	89%	82%	92%	92%	100%	
***C*** **. *mzymtae* Sanger**	99%	98%	93%	86%	98%	97%	93%	100%

Note

The similarity between the reads ranges from 83% to 88% between the *C*. *insignis* reads and 89% to 93% between the *C*. *mzymtae* reads for the High Accuracy basecalled reads. Between those two species, the similarity of the raw reads varies between 83 and 92%. For the Super Accuracy basecalled reads (which are the same individual reads as the High Accuracy reads), those similarities are somewhat higher which can explain the slightly better results in Table 7. The top and bottom rows show the similarity of the raw reads with the Sanger reference of *C*. *insignis* and *C*. *mzymtae*. (HAC: High Accuracy, SupHAC: Super Accuracy, *C*.: *Cordulegaster*).

**TABLE A7:** Memory usage of Amplicon_sorter when analyzing big datasets with different number of species

Dataset	# Reads used	Amplicon length	# Species	Peak memory usage (GB)
Own dataset	142,000	600–5000	50	7
Srivathsan et al. (2021b) Mixed Diptera	100,000	658	511	8
Srivathsan et al. (2021b) Mixed Diptera	300,000	658	511	17
Srivathsan et al. (2021b) Mixed Diptera	568,000	658	511	70
Srivathsan et al. (2021b) Palaearctic Phoridae	100,000	658	9929	14
Srivathsan et al. (2021b) Palaearctic Phoridae	200,000	658	9929	19
Srivathsan et al. (2021b) Palaearctic Phoridae	500,000	658	9929	85

Note

With 50 species in the sample and several amplicons, the memory usage does not exceed 7 GB. With increased number of species in the sample and increased number of reads sampled, the peak memory utilization increased drastically.
